# Genomic characterization of mobile genetic elements associated with antimicrobial resistance in Streptococcus pneumoniae from Australia

**DOI:** 10.1099/mgen.0.001662

**Published:** 2026-04-08

**Authors:** Gabriel Temitope Sunmonu, Rosa C. Coldbeck-Shackley, Rikki M. A. Graham, Lex EX Leong, Abiodun David Ogunniyi, Anna E. Sheppard

**Affiliations:** 1 School of Animal and Veterinary Sciences, Adelaide University, Roseworthy, South Australia 5371, Australia; 2 Australian Centre for Antimicrobial Resistance Ecology, Adelaide University, Adelaide, South Australia, Australia; 3 Public Health Laboratory, Microbiology and Infectious Diseases, SA Pathology, Adelaide, South Australia, Australia; 4 Queensland Department of Health, Public Health Microbiology, Public and Environmental Health Reference Laboratories, Brisbane, Queensland, Australia; 5 School of Biological Sciences, Adelaide University, Adelaide, South Australia 5005, Australia

**Keywords:** antimicrobial resistance, integrative conjugative elements, mobile genetic elements, multidrug resistance, *Streptococcus pneumoniae*

## Abstract

The emergence and spread of antimicrobial resistance (AMR) in *Streptococcus pneumoniae* threatens current antibiotic treatment strategies. While *β*-lactams remain the first-line therapy for pneumococcal infections in Australia, resistance to macrolides, tetracyclines and other antibiotics, driven by resistance genes carried on mobile genetic elements (MGEs), is increasingly reported. In this study, we conducted a comprehensive analysis of 573 *S*. *pneumoniae* genomes from South Australia, Queensland and Victoria to investigate the distribution of MGEs and their association with acquired AMR genes. Resistance genes and MGEs were identified using AMRFinderPlus and MobileElementFinder. Serotypes, sequence types and global pneumococcal sequence clusters (GPSCs) were assigned using SeroBA, MLST and the GPS pipeline. Out of the 573 genomes, 547 passed quality checks. Tn*916*-like (Tn*916*, Tn*6002*, Tn*2010*, Tn*6003* and ICE*Spn*Tw19F14) and Tn*5253*-like (Tn*5253*, ICE*Spn*529IQ) integrative conjugative elements carried various combinations of *erm*B, *mef*A, *msr*D, *tet*M, *cat*A and *cat*A16 genes, supporting horizontal gene transfer as a key mechanism of resistance spread. Macrolide and tetracycline resistance genes co-occurred in 192/239 (80.7%) MGE-positive genomes. The most common MGE-positive serotypes were 33F/ST717/GPSC3 (15.6%, *n*=30), serotype 4/ST2759/GPSC162 (15.1%, *n*=29), serotype 15A/ST63/GPSC9 (7.3%, *n*=14), serotype 23A/ST338/GPSC5 (5.7%, *n*=11), serotype 15A/ST8625/GPSC9 (3.6%, *n*=7) and serotype 19A/ST3111/GPSC932 (3.6%, *n*=7). Our results reflect global trends of MGE-associated resistance in expanding non-vaccine serotypes (such as 15A and 23A) and multidrug-resistant clones. These findings underscore the evolutionary role of MGEs associated with AMR in shaping the pneumococcal resistome and highlight the continuous need for genomic surveillance to inform antibiotic stewardship and vaccine strategies in Australia.

**Impact Statement**

The global rise of antimicrobial resistance (AMR) in *Streptococcus pneumoniae* continues to threaten treatment efficacy and public health interventions. Using whole-genome sequence data across South Australia, Queensland and Victoria, we characterized the distribution of mobile genetic elements (MGEs) associated with acquired resistance determinants. Our analysis identified Tn*916*-like and Tn*5253*-like integrative conjugative elements as major vectors mobilizing macrolide and tetracycline resistance genes, with Tn*6002* predominating. MGE-associated resistance was frequently detected in expanding non-vaccine serotypes, particularly 15A and 23A, reflecting global shifts towards multidrug-resistant, non-vaccine lineages. These findings underscore the evolutionary significance of MGEs in shaping the pneumococcal resistome and the ongoing diversification of resistant clones. Continued genomic surveillance and the development of serotype-independent vaccines will be critical to mitigating pneumococcal disease and the spread of AMR in *S. pneumoniae*.

## Data Summary

Genome sequences are publicly available in the National Center for Biotechnology Information (NCBI) under BioProject PRJNA1125055 for South Australia, BioProject PRJEB97785 for Queensland and BioProject PRJNA857543 for Victoria. Accession numbers for each sequence data are listed in Table S1.

## Introduction

*Streptococcus pneumoniae* (the pneumococcus) is an opportunistic pathogen that asymptomatically colonizes the upper respiratory tract [1]. *S. pneumoniae* is the leading cause of vaccine-preventable death in developing countries [2]. It mostly affects children (under 5 years) and the elderly (over 65 years). When the body’s defence is compromised and immunity is low, depending on the serotype, *S. pneumoniae* can cause otitis media (middle ear infection), pneumonia (lung inflammation), bacteraemia (blood infection) and meningitis (brain inflammation) [1,3, 4]. The capsular polysaccharide of *S. pneumoniae*, synthesized by proteins encoded within the capsular polysaccharide (*cps*) locus, is essential for the virulence of the organism. To date, over 100 serotypes of *S. pneumoniae* have been identified, distinguished by differences in the structure of their capsular polysaccharide [5,6].

The introduction of pneumococcal conjugate vaccines (PCVs) has significantly reduced the global incidence of invasive pneumococcal disease (IPD) by targeting the most prevalent disease-associated serotypes [7,8]. In Australia, PCVs have been part of the national immunization schedule since 2005, with broader coverage achieved through successive vaccines protecting against more serotypes [9,10]. Despite these efforts, IPD remains a public health concern, with incidence continuing to rise in recent years both globally and in Australia [11,15]. In Australia, many high-risk individuals, especially Indigenous Australians, still face barriers to accessing vaccines. Data from the Department of Health and Aged Care’s National Notifiable Disease Surveillance System show that the incidence of IPD has reached its highest level since 2004 (*n*=2,355), with cases rising from 1,858 in 2022 to 2,268 in 2023 and 2,378 in 2024 [14,15].

*S. pneumoniae* treatment in Australia varies by infection severity and patient factors [16]. Mild community-acquired pneumonia requires high-dose oral amoxicillin as first-line therapy, or macrolides for penicillin-allergic patients. Moderate–severe cases require hospitalization and intravenous therapy with *β*-lactams (benzylpenicillin, ceftriaxone and cefotaxime), often plus macrolides. In cases of other IPD such as meningitis, ceftriaxone/cefotaxime is the main treatment choice, with vancomycin added if resistant strains are suspected [17]. Severe penicillin allergy patients receive macrolides or doxycycline [16]. While vaccination and antibiotic therapy have complemented each other in managing pneumococcal infections, resistance to antibiotics has emerged as an ongoing challenge [18]. Notably, penicillin-resistant *S. pneumoniae* has become increasingly prevalent worldwide [19,20]. Resistance to other classes, including macrolides and tetracyclines, has also been reported with rising frequency across global settings [21,24].

Antibiotic resistance in *S. pneumoniae* can occur via chromosomal mutations or horizontal gene transfer (HGT). Mutations in penicillin-binding proteins (*pbp2x*, *pbp2b *and *pbp1a*) reduce the affinity of *β*-lactams for their targets. Changes in ribosomal proteins (L4 and L22) or in 23S rRNA interfere with macrolide binding [25], while mutations in DNA gyrase/topoisomerase IV confer resistance to fluoroquinolones. In addition, *S. pneumoniae* can acquire resistance through HGT. These include *erm*B, which methylates 23S rRNA, and *mef*A/E, which encodes macrolide efflux pumps. Other examples are *cat*A, responsible for chloramphenicol acetylation, and *tet*M, which encodes a ribosomal protection protein conferring tetracycline resistance [26].

The pneumococcus is naturally competent, capable of acquiring exogenous genetic material. This genetic plasticity facilitates the uptake and dissemination of antimicrobial resistance (AMR) determinants via HGT [27]. Mobile genetic elements (MGEs), particularly plasmids and integrative conjugative elements (ICEs), play a central role in the mobility of resistance genes within and between bacterial populations. While plasmids are infrequently detected in *S. pneumoniae* [28], several ICEs associated with AMR in *S. pneumoniae* have been well characterized [13]. Among the earliest identified were the ICEs Tn*916* [29] and Tn*5253* [30]. Tn*916*-like elements commonly carry tetracycline and macrolide resistance genes, whereas Tn*5253*-like elements are typically associated with resistance to tetracycline and chloramphenicol [31,33].

Using a dataset of 573 pneumococcal genomes from three Australian states (South Australia, Queensland and Victoria), we identified AMR genes circulating and their association with MGEs within these genomes.

## Methods

### Study design

A total of 573 sequenced *S. pneumoniae* genomes from pneumococcal disease cases in South Australia (*n*=246; BioProject PRJNA1125055), Queensland (*n*=163; BioProject PRJEB97785) and Victoria (*n*=164; BioProject PRJNA857543), collected between 2018 and 2024, were analysed in this study (Table S1, available in the online Supplementary Material). Genomes from South Australia and Queensland were generated using the Illumina NextSeq 500, with libraries prepared using the Nextera XT library prep kit (Illumina, USA) [34]. The dataset for Victoria was a subset from a recent study of *S. pneumoniae* collected between 2018 and 2022 from Victoria, Australia [35]. That study reported the serotype, global pneumococcal sequence cluster (GPSC), sequence type (ST) distribution and AMR profiles of the isolates but did not investigate the association of MGEs with the identified AMR genes. The authors identified 165 genomes with acquired AMR genes; we used slightly different methods for AMR gene detection, which identified AMR determinants in 164 of these genomes. These were included in this study for MGE analysis to investigate the potential association between acquired resistance and MGEs (Table S1).

### Genome quality control and assembly

Raw reads were processed using the Global Pneumococcal Sequencing (GPS) pipeline (v. 1.0.0) [36], which included genome assembly using Shovill (https://github.com/tseemann/shovill) (v. 1.1.0). Quality control was performed using default thresholds. A total of 547 genomes (South Australia: *n*=233; Queensland: *n*=150; and Victoria: *n*=164) passed quality checks and were used for downstream analysis.

### Serotyping, multi-locus sequence typing and GPSC assignment

Within the GPS pipeline, *in silico* serotyping was performed using seroBA (v. 2.0.4) [37]. Multi-locus sequence typing (MLST) was carried out using MLST (v. 2.23.0) (https://github.com/tseemann/mlst) with reference to the *S. pneumoniae* profile in the PubMLST database (https://pubmlst.org/) [38]. GPSCs were assigned using PopPUNK (v. 2.6.3) [39] and the GPSC database (GPSC_v9) [40], employing a *k*-mer-based clustering approach.

### Detection of AMR genes and MGEs

MGEs were identified by performing blastn (v. 2.14.1) [41] searches of the assembled genomes against the MobileElementFinder database (v. 1.0.2) [42] using a 95% identity threshold. In cases where multiple MGEs aligned to the same genomic region, only the MGEs associated with AMR (AMR-MGEs) with the highest alignment length and bit score were retained through manual filtering. An MGE was considered to be present if the retained alignments covered >90% of the MGE length. Additional blast searches were conducted on *tet*32-positive genomes against a 50 kb genomic island associated with *tet*32 [22] and on *mef*A- and *msr*D-positive genomes against the macrolide efflux genetic assembly (MEGA) element (accession no. AF274302) using 95 and 90% identity thresholds, respectively, to confirm the presence of these MGEs.

In order to minimize artefactual discrepancies between AMR and MGE prediction, AMR genes were predicted from the assembled genomes using AMRFinderPlus (v. 4.0.23) [43]. We chose this tool because it uses genome assemblies as input, as is the case for the MGE prediction tool, MobileElementFinder. This contrasts with ARIBA, which is integrated into the GPS pipeline and operates on FASTQ reads. Genomes carrying acquired AMR genes were inferred to be resistant to the corresponding antibiotics. We employed the GPS pipeline to infer penicillin resistance because it identifies the different alleles of each *pbp* gene (*pbp1a*, *pbp2b* and *pbp2x*). The presence of individual *pbp* genes alone does not necessarily translate into phenotypic penicillin resistance; rather, resistance requires specific combinations of *pbp* gene alleles.

### Data visualization

Figures were created in R (v. 4.4.3) (https://www.R-project.org/) using the Tidyverse suite [44] along with ggbreak [45] and UpSetR [46]. The identified AMR-MGEs were annotated using bakta web v. 1.11.3 [47,48] and visualized using LoVis4u [49].

## Results

### AMR in *S. pneumoniae*

Among the 383 sequenced *S. pneumoniae* genomes from South Australia and Queensland, 30% (*n*=115) were predicted to be resistant to at least one antibiotic. Using meningitis breakpoints from the GPS pipeline, 17.2% (*n*=66) were predicted to be resistant to penicillin. Sixty-eight genomes (17.8%) carried at least one acquired macrolide resistance gene. *erm*B was the most common (*n*=54), followed by *mef*A (*n*=20) and *msr*D (*n*=20). All of the genomes carrying *mef*A also carried *msr*D, and six genomes carried all three macrolide resistance genes. Sixty-seven genomes (17.5%) carried at least one acquired tetracycline resistance gene, most commonly *tet*M (*n*=65), while *tet*32, an acquired tetracycline resistance gene typically associated with a ~50 kb genomic island, was detected in two genomes. Two genomes carried chloramphenicol resistance genes *cat*A (*n*=1) and *cat*A16 (*n*=1) on a Tn*5253*-like element (Table S1).

**Fig. 1.**
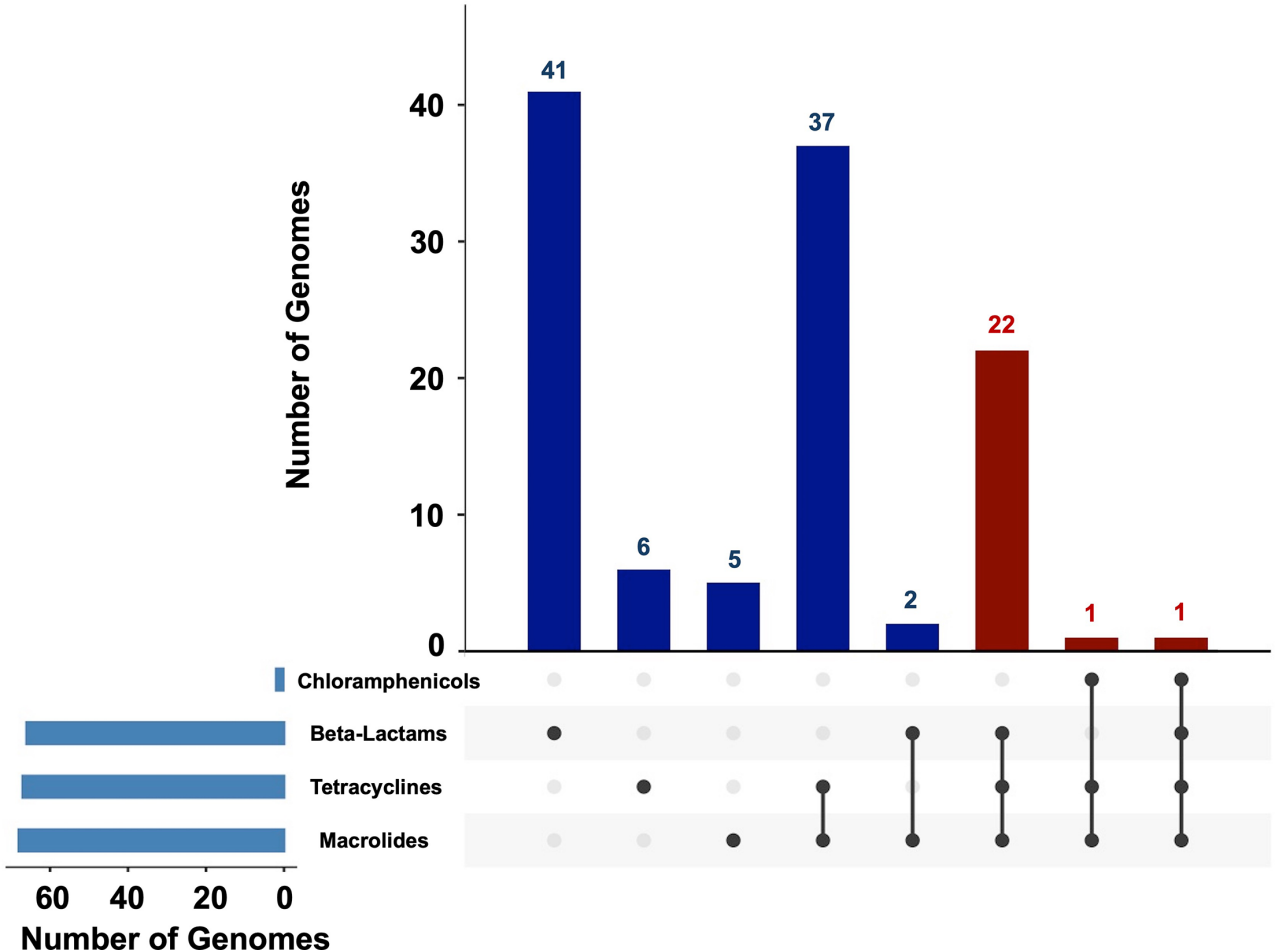
Upset plot showing the distribution of genomically inferred AMR classes of *S. pneumoniae* genomes from South Australia and Queensland. Inferred MDR genomes are highlighted in red.

Genomically inferred resistance to different antibiotic classes is presented in Fig. 1. Inferred resistance to macrolides was present in 68 genomes, followed closely by tetracycline (*n*=67), beta-lactams (*n*=66) and chloramphenicol (*n*=2). In terms of occurrence, inferred resistance to beta-lactams alone was the most prevalent (*n*=41), followed by the combination of tetracycline and macrolide (*n*=37) and then the combination of beta-lactam, tetracycline and macrolide (*n*=22). Inferred multidrug resistance (MDR; resistance to three or more classes of antibiotics) was observed in 24 genomes (Fig. 1). The MDR serotypes included 15B (*n*=5), 15A (*n*=4), 19A (*n*=3), 19F (*n*=3), 23A (*n*=3), 15C (*n*=2), 3 (*n*=1), 6D (*n*=1), 6E (*n*=1) and non-typable (*n*=1).

### MGE distribution and association with AMR genes

**Fig. 2.**
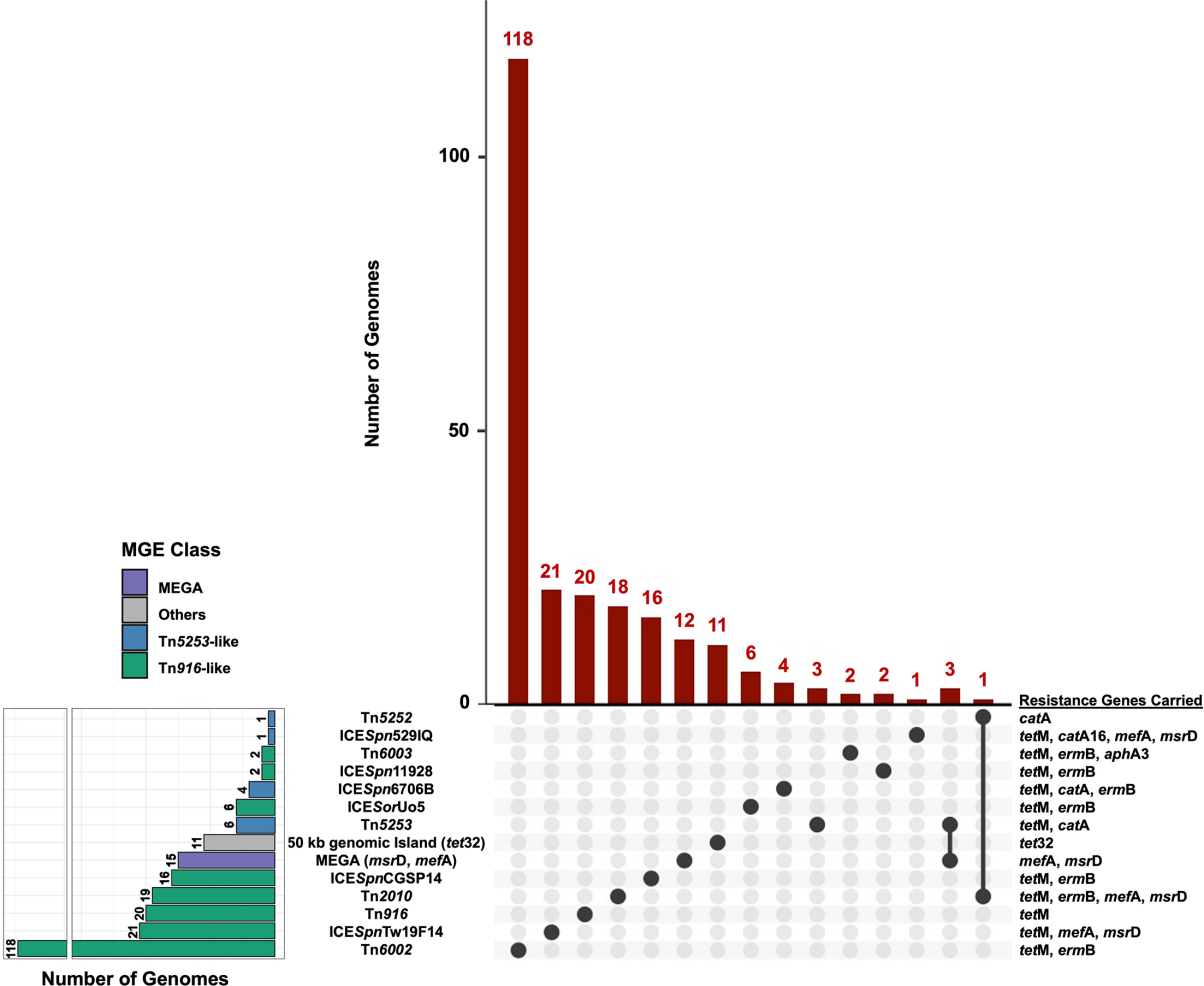
Upset plot illustrating the distribution of AMR-MGEs in *S. pneumoniae* genomes from South Australia, Queensland and Victoria. Co-occurrence of MEGA with Tn*5253* (*n*=3) and Tn*2010* with Tn*5252* (*n*=1) is also shown.

We identified AMR-MGEs among genomes carrying acquired AMR determinants (*erm*B, *mef*A, *msr*D, *tet*M, *tet*32, *cat*A, *cat*A16 and *aphA*3-IIIa) from South Australia, Queensland and Victoria. AMR-MGEs were present in 238 genomes: South Australia (*n*=49), Queensland (*n*=25) and Victoria (*n*=164) (Table S2). blast analysis revealed *tet*32-positive genomes with ≥99.98% identity to a previously described 50 kb genomic island from *S. pneumoniae* [22]. In each case, the coordinates of *tet*32 fell within the genomic island boundaries, suggesting that this element is conserved in our dataset. The most common AMR-MGEs were Tn*916*-like elements (85.7%, *n*=204), followed by MEGA (6.3%, *n*=15), Tn*5253*-like elements (5%, *n*=12) and the 50 kb genomic island (4.6%, *n*=11) associated with *tet*32 [22] (Fig. 2). MEGA co-occurred with Tn*5253* in three genomes, while a Tn*916*-like (Tn*2010*) element co-occurred with a Tn*5253*-like element (Tn*5252*) in one genome.

**Fig. 3.**
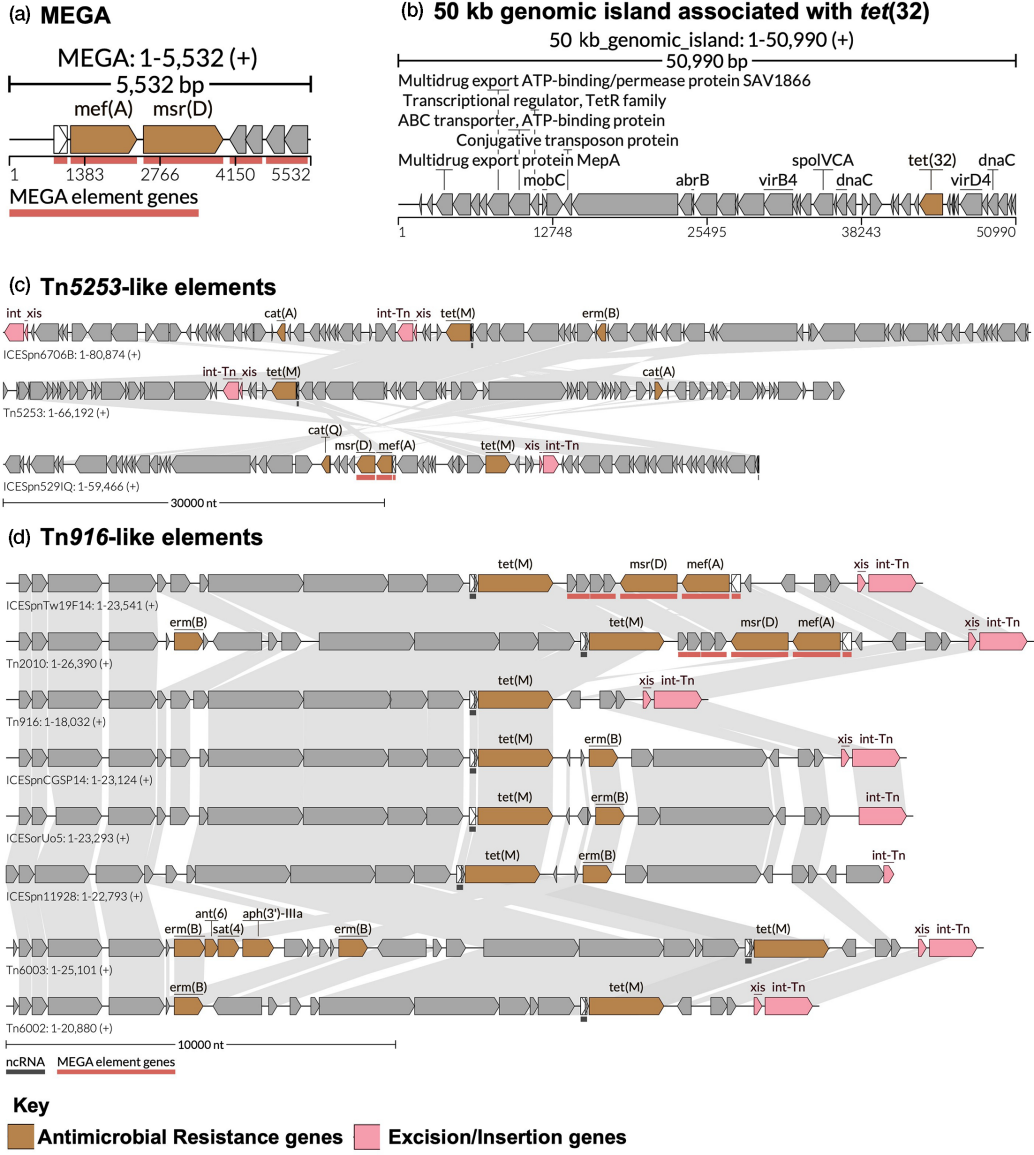
A schematic diagram of MGEs associated with AMR in *S. pneumoniae*. Structure of (**a**) MEGA, (**b**) 50 kb genomic island associated with *tet*32, (**c**) Tn*5253*-like elements and (**d**) Tn*916*-like elements in this study is shown with AMR genes in orange colour, while excision and insertion genes are in peach colour.

Overall, the most common element was Tn*6002* (49.6%, *n*=118), followed by ICE*Spn*Tw19F14 (8.8%, *n*=21), Tn*916* (8.4%, *n*=20), Tn*2010* (8%, *n*=19), ICE*Spn*CGSP14 (6.7%, *n*=16), MEGA (6.3%, *n*=15), 50 kb genomic island (4.6%, *n*=11), ICE*Sor*Uo5 (2.5%, *n*=6), Tn*5253* (2.5%, *n*=6), ICE*Spn*6706B (1.7%, *n*=4), ICE*Spn*11928 (0.8%, *n*=2), Tn*6003* (0.8%, *n*=2), ICE*Spn*529IQ (0.4%, *n*=1) and Tn*5252* (0.4%, *n*=1). The identified MGEs harboured various combinations of resistance genes (Fig. 3), which were the same in every genome carrying the same MGE, as detailed in Fig. 2.

Analysis of the assembled contigs revealed that acquired AMR genes were co-located within MGEs. Specifically, the resistance genes were always found on the same contigs as their associated MGEs, with their genomic coordinates falling entirely within the boundaries of the respective MGEs (Table S2). This genomic context strongly suggests that these MGEs serve as vehicles for the acquisition and dissemination of acquired AMR determinants in *S. pneumoniae*, reinforcing their role in HGT events contributing to resistance evolution.

### Serotype, STs and GPSC distribution

**Fig. 4.**
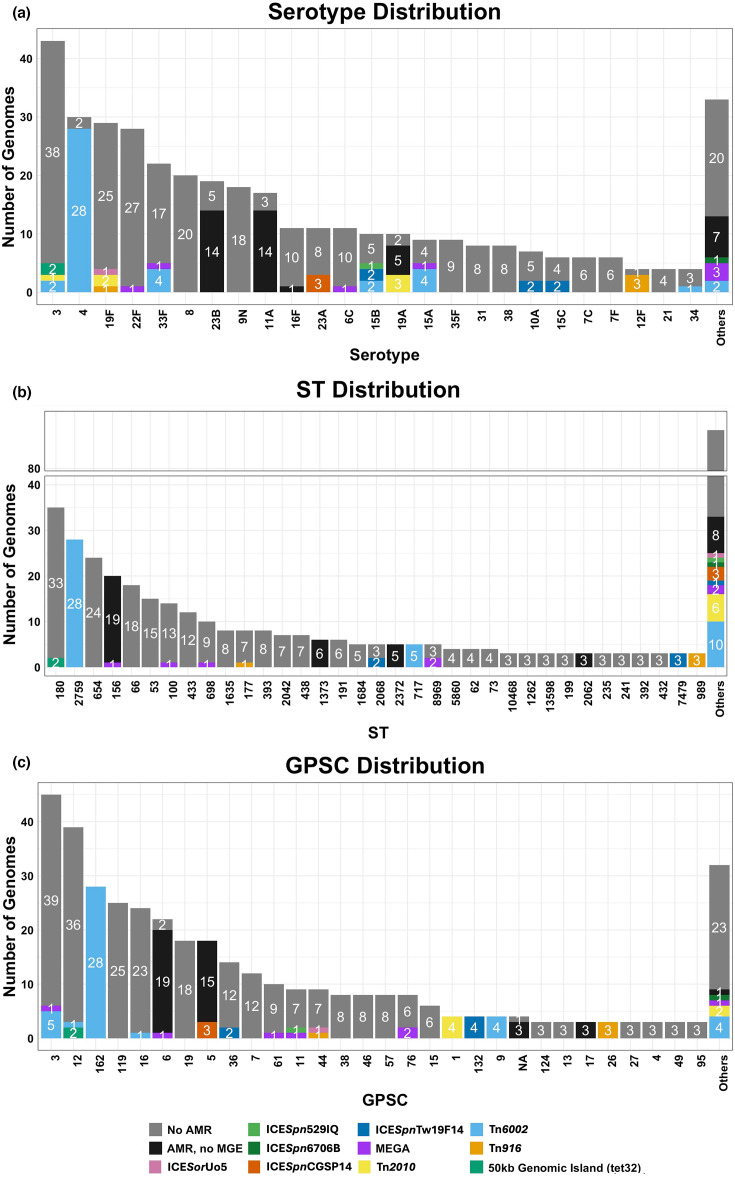
Distribution of AMR-MGEs within *S. pneumoniae* genomes from South Australia and Queensland based on (**a**) serotype, (**b**) ST and (**c**) GPSC. The distribution is coloured based on genomes with inferred AMR. Genomes with inferred resistance without AMR-MGE are shown as ‘AMR, no MGE’ (black), while genomes with no inferred resistance are shown as ‘no AMR’ (grey). AMR-MGEs are represented as ICE*Sor*Uo5 (pink), ICE*Spn*529IQ (green), ICE*Spn*6706B (dark green), ICE*Spn*CGSP14 (orange), ICE*Spn*Tw19F14 (blue), MEGA (purple), Tn*2010* (yellow), Tn*6002* (light blue), Tn*916* (orange-yellow) and 50 kb genomic island (tet32) (teal).

Out of the 383 sequenced *S. pneumoniae* genomes from South Australia and Queensland, 44 distinct serotypes were identified (Fig. 4a, Table S1). The most prevalent serotypes were 3 (11.2%, *n*=43), 4 (7.8%, *n*=30), 19F (7.6%, *n*=29), 22F (7.3%, *n*=28), 33F (5.7%, *n*=22) and 8 (5.2%, *n*=20). MGEs containing AMR genes were detected in 19.3% (*n*=74) of these, with the most common serotypes carrying them being serotype 4 (93%, *n*=28), serotype 3 (11.6%, *n*=5), serotype 15A (55.6%, *n*=5), serotype 15B (50%, *n*=5) and serotype 33F (22.7%, *n*=5). Genomes with inferred resistance (such as beta-lactam resistance) that were not associated with AMR-MGEs were most common among serotypes 11A (82.4%, *n*=14), 23B (73.7%, *n*=14) and 19A (50%, *n*=5). In contrast, serotypes 8 (*n*=20) and 9N (*n*=18) were the most common serotypes with no inferred resistance.

The 383 genomes comprised 108 unique STs (Fig. 4b), with a small number of dominant lineages accounting for a substantial proportion of the dataset. ST180, ST2759, ST654, ST156 and ST66 were the most prevalent STs overall. Notably, all isolates belonging to ST2759 carried at least one acquired AMR gene within an MGE, whereas other common STs, including ST654, ST66, ST53 and ST433, lacked inferred resistance.

Using the GPSC framework, we identified 55 distinct GPSCs (Fig. 3c). GPSC3, GPSC12, GPSC162, GPSC119 and GPSC16 were the most common clusters. For certain GPSCs, including GPSC162, all isolates carried acquired AMR genes within an MGE, while other prevalent GPSCs, such as GPSC119 and GPSC19, had no isolates with inferred resistance.

Among the South Australian and Queensland datasets, several dominant serotype/ST/GPSC combinations were observed, most notably serotype 3/ST180/GPSC12 and serotype 4/ST2759/GPSC162, with additional common combinations shown in Fig. S1.

The serotype, ST and GPSC distribution of the Victoria dataset is provided in Table S1. These data are not presented here as they have been previously reported by Higgs *et al.* [35].

### MGE association with serotypes, GPSCs and STs

**Fig. 5.**
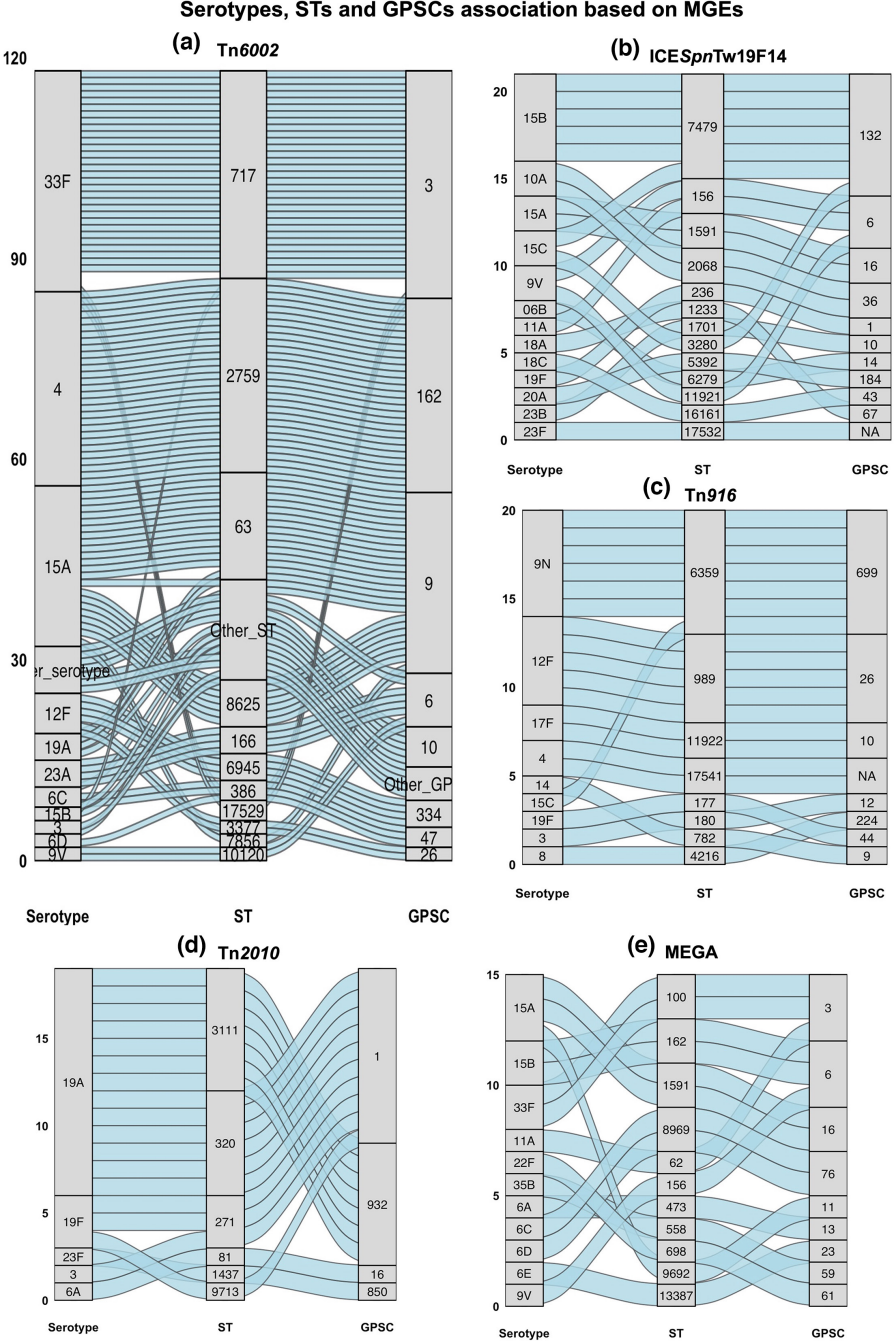
Alluvial plot showing associations between serotypes, STs and GPSCs carrying MGEs [(a) Tn*6002*, (b) ICE*Spn*Tw19F14, (c) Tn*916*, (d) Tn*2010* and (e) MEGA] from South Australia, Queensland and Victoria. Serotypes, STs and GPSCs represented by fewer than two genomes are grouped as ‘Others’ in (a).

The correlation of AMR-MGEs with serotype/ST/GPSC among the 238 MGE-positive genomes from South Australia, Queensland and Victoria is shown in Fig. 5 for AMR-MGEs with more than 5 serotype/ST/GPSC combinations. The most common AMR-MGE detected was Tn*6002* (*n*=118), which was identified in 18 serotypes, 26 STs and 13 GPSCs (Fig. 5a). The predominant serotype/ST/GPSC combinations carrying Tn*6002* were serotype 33F/ST717/GPSC3 (*n*=30), serotype 4/ST2759/GPSC162 (*n*=29) and serotype 15A/ST63/GPSC9 (*n*=14).

The second most common AMR-MGE was ICE*Spn*Tw19F14 (*n*=21), detected in 13 serotypes, 13 STs and 11 GPSCs (Fig. 5b). These included serotype 15B/ST7479/GPSC132 (*n*=5), serotype 10A/ST2068/GPSC36 (*n*=2) and serotype 15A/ST1591/GPSC16 (*n*=2). Tn*916* (*n*=20) was present in nine serotypes, eight STs and eight GPSCs, including serotype 9 N/ST6359/GPSC699 (*n*=6) and serotype 12F/ST989/GPSC26 (*n*=5) (Fig. 5c). Tn*2010* (*n*=19) was identified in five serotypes, six STs and four GPSCs, comprising serotype 19A/ST3111/GPSC932 (*n*=7), serotype 19A/ST320/GPSC1 (*n*=6) and serotype 19F/ST271/GPSC1 (*n*=2) (Fig. 5d).

ICE*Spn*CGSP14 (*n*=16) occurred in three serotypes, four STs and two GPSCs, including serotype 23A/ST338/GPSC5 (*n*=11) and serotype 23A/ST5242/GPSC5 (*n*=2). ICE*Sor*Uo5 (*n*=6) was detected in two serotypes, three STs and two GPSCs: serotype 19A/ST2013/GPSC10 (*n*=3), serotype 19F/ST179/GPSC44 (*n*=2) and serotype 19F/ST17368/GPSC44 (*n*=1). ICE*Spn*11928 (*n*=2) was found exclusively in serotype 19F/ST179/GPSC44 (*n*=2). Tn*6003* (*n*=2) occurred in two serotypes, two STs and two GPSCs: serotype 19A/ST276/GPSC10 and serotype 14/ST143/GPSC6.

Tn*5253* (*n*=6) was identified in four serotypes, four STs and four GPSCs, including serotype 12F/ST13456/GPSC26 (*n*=2) and serotype 15A/ST1591/GPSC16 (*n*=2). ICE*Spn*6706B (*n*=4) occurred in three serotypes, three STs and three GPSCs including serotype 3/ST180/GPSC12 (*n*=2), serotype 12F/ST989/GPSC26 (*n*=1) and serotype 6E/ST95/GPSC23 (*n*=1). ICE*Spn*529IQ (*n*=1) was detected only in serotype 15B/ST14013/GPSC11 and Tn*5252* (*n*=1) only in serotype 6A/ST81/GPSC16.

MEGA (*n*=15) was present in 11 serotypes, 13 STs and 9 GPSCs, including serotype 15A/ST1591/GPSC16 (*n*=2), serotype 15B/ST162/GPSC6 (*n*=2) and serotype 33F/ST100/GPSC3 (*n*=2) (Fig. 5e). The 50 kb genomic island (*n*=11) was exclusively associated with serotype 3/ST180/GPSC12 (*n*=11).

Overall, several AMR-MGEs appeared to be strongly associated with specific serotype/ST/GPSC lineages, consistent with lineage fixation in many cases. However, variation in MGE carriage was observed within selected lineages. Notably, within serotype 3/ST180/GPSC12, multiple MGEs were detected, including ICESpn6706B and a 50 kb genomic island, while a subset of isolates lacked detectable AMR-MGEs. Similarly, within serotype 33F/ST100/GPSC3, the MEGA element was present in only a minority of isolates, with the remainder carrying no identifiable AMR-MGE. These findings indicate that while MGE-lineage associations are often stable, they are not universally fixed.

## Discussion

Globally, the incidence of pneumococcal disease declined following the introduction of PCVs. However, it is now rising, largely due to serotype replacement and vaccine failure [13,50,52]. In Australia, despite the implementation of higher-valent PCVs, the burden of pneumococcal disease has recently increased [14]. This resurgence is likely driven by infections caused by non-vaccine serotypes (serotype replacement) or vaccine-protected serotypes that evade immunity (vaccine failure) [35,53]. The challenge is further complicated by infections involving AMR or MDR *S. pneumoniae*, which undermine the complementary role of antibiotics in pneumococcal disease management.

Although previous Australian studies have examined AMR in *S. pneumoniae* [54,56], few have characterized resistance determinants [35], and most investigations of MGEs rely on molecular methods such as PCR [57]. In this study, we utilized whole-genome sequencing to investigate MGEs associated with acquired AMR genes in *S. pneumoniae* genomes from South Australia, Queensland and Victoria.

The most prevalent serotypes identified in South Australia and Queensland (3, 4, 22F and 19F) are similar to those reported in previous Australian (Victoria) and Asia-Pacific studies, with the exception of serotype 4 [12,35, 58]. Our findings reveal that macrolide resistance in *S. pneumoniae* remains a key concern, with 17.8% (68/383) of genomes from South Australia and Queensland harbouring at least one acquired macrolide resistance gene. This is slightly higher than the 11.3% (145/1286) erythromycin resistance rate previously reported in Victoria [35]. The most common determinant was *erm*B, often co-located with *tet*M on Tn*916*-like elements. Notably, macrolide-resistant pneumococci are prioritized on the WHO’s global pathogen watchlist [59] due to their increasing clinical significance and limited treatment options, particularly in low-resource settings. While *β*-lactams remain the primary clinical therapeutic agents of treatment in Australia, our results underscore the concerning potential of macrolide resistance. This is consistent with other Australian studies [35,56], though most lacked detailed characterization of MGEs. Globally, macrolide resistance is more prevalent in regions with high macrolide consumption [24,60, 61], but Australia presents a unique paradox with resistance occurring even amid lower clinical [62] and community [63] usage. This is likely due to historical use or MGE-mediated co-selection, where other resistance determinants are carried on the same MGE, a pattern consistent with global genomic observations [64,66].

Among MGE-positive genomes from South Australia, Queensland and Victoria, serotypes 33F, 4, 15A, 19A, 23A, 3 and 12F were the most frequently observed. These trends are consistent with global reports [67,70] that highlight increasing prevalence of non-vaccine serotypes like 15A and 23A, which are often MDR and carry Tn*916*-like elements. Globally, serotype 15A has emerged as a significant MDR clone in the post-PCV13 era, frequently linked with these AMR-MGEs [13,23, 71].

At the clonal level, we observed disproportionate MGE carriage among specific STs. All ST2759, ST717, ST7479 and ST989 carried MGEs, whereas commonly circulating STs such as ST654, ST66 and ST53 have no AMR. This pattern indicates expansion of MGE-harbouring clones that likely play a pivotal role in resistance gene dissemination. ST2759 appears to be a key driver of resistance in our study, consistent with North American findings where this ST forms part of internationally disseminated MDR lineages [72].

Based on the GPSC classification, all genomes assigned to GPSC162, GPSC132, GPSC9, GPSC1 and GPSC26 carried MGEs, highlighting strong clonal associations with AMR acquisition. GPSC162 (corresponding to serotype 4/ST2759) was the dominant MGE-carrying lineage, reinforcing its public health significance as previously reported in the Canadian SAVE study [72]. Similarly, most GPSC26 (serotype 12F/ST989) harboured MGEs. In contrast, common Australian GPSCs such as GPSC7, GPSC19, GPSC38 and GPSC119 have no AMR in our dataset, suggesting that they may not currently contribute to AMR dissemination. This finding is similar to those of Lo *et al.* [73], though their potential for future acquisition remains unknown.

The majority of MGEs detected were Tn*916*-like elements (*n*=204), notably Tn*6002*, ICE*Spn*Tw19F14, Tn*916* and Tn*2010*, which carried various combinations of *tetM*, *ermB*, *mefA* and *msrD*. Tn*5253*-like elements were less frequent but notable for carrying *cat*A or *catA*16, conferring resistance to chloramphenicol, which remains important for treatment in low-income settings like Papua New Guinea [74] which is a close neighbour to Australia.

Our findings confirm the 50 kb genomic island carrying *tet*32 in Australian *S. pneumoniae* genomes, indicating its stability and geographic spread beyond the UK *S. pneumoniae* isolate described by Nikolaou *et al*. [22]. The ≥99.98% sequence identity shows the genomic island is highly conserved and likely a recent acquisition in pneumococci. Its mobility and replication-associated genes, along with homology to elements in *Parvimonas micra* and *Streptococcus agalactiae*, support an origin from conjugative plasmids in oral bacteria [22]. Its persistence underscores the role of HGT in sustaining tetracycline resistance, particularly with ongoing clinical and veterinary use. Detection of the *tet32*-carrying genomic island across *S. pneumoniae* genomes from multiple Australian states suggests that it is not sporadic but part of the expanding pneumococcal resistome. Ongoing genomic surveillance is needed to track its distribution, recombination with other mobile elements and impact on resistance evolution in *S. pneumoniae*.

Compared to previous Australian studies that used PCR-based methods, our genomic approach allowed for comprehensive characterization of the structural context of MGEs, confirming that AMR genes were embedded within defined MGE boundaries. This genomic resolution reinforces the role of HGT as a key mechanism driving resistance dissemination in *S. pneumoniae* in Australia. Globally, the prevalence of Tn*916*-like elements among pneumococcal genomes is well-documented [21,23, 58, 65], especially in MDR lineages such as GPSC6 [75], underscoring the conserved and critical role of these MGEs in AMR spread.

## Conclusion

To date, this study provides the most comprehensive genomic characterization of MGEs associated with acquired AMR in *S. pneumoniae* from Australia. Our findings demonstrate that MGEs, particularly Tn*916*-like elements, are likely the primary vectors mediating the dissemination of macrolide and tetracycline resistance in our dataset. These AMR-MGEs show strong association with specific serotypes, STs and GPSCs, indicating clonal expansion of resistant lineages. The co-localization of AMR genes within MGE boundaries confirms the central role of HGT in shaping the pneumococcal resistome. While our dataset represents a large and diverse collection, it does not capture all *S. pneumoniae* circulating across Australia, and this should be considered when interpreting the findings. Continued genomic surveillance integrating AMR-MGE profiling is essential to detect emerging high-risk resistant clones and to inform vaccine and treatment strategies in the evolving post-PCV era. Our findings also reinforce the need for continuing efforts towards the development of a broadly protective, capsular-independent vaccine to prevent pneumococcal disease and simultaneously reduce the incidence of AMR.

## Supplementary material

10.1099/mgen.0.001662 Uncited Fig. S1.

10.1099/mgen.0.001662 Uncited Table S1.

## Abbreviations

AMR: antimicrobial resistance
GPS: Global Pneumococcal Sequencing
GPSC: global pneumococcal sequence cluster
HGT: horizontal gene transfer
ICE: integrative conjugative element
IPD: invasive pneumococcal disease
MDR: multidrug resistance
MEGA: macrolide efflux genetic assembly
MGE: mobile genetic element
PCV: pneumococcal conjugate vaccine
ST: sequence type
