# Supplementary material for: Genomic characterization of mobile genetic elements associated with antimicrobial resistance in Streptococcus pneumoniae from Australia
Source: Microb Genom. 2026 Apr 8;12(4):001662. doi: 10.1099/mgen.0.001662 (PMC13085319; doi:10.1099/mgen.0.001662)
Supplement: Uncited Fig. S1. [file mgen-12-01662-s001.pdf]

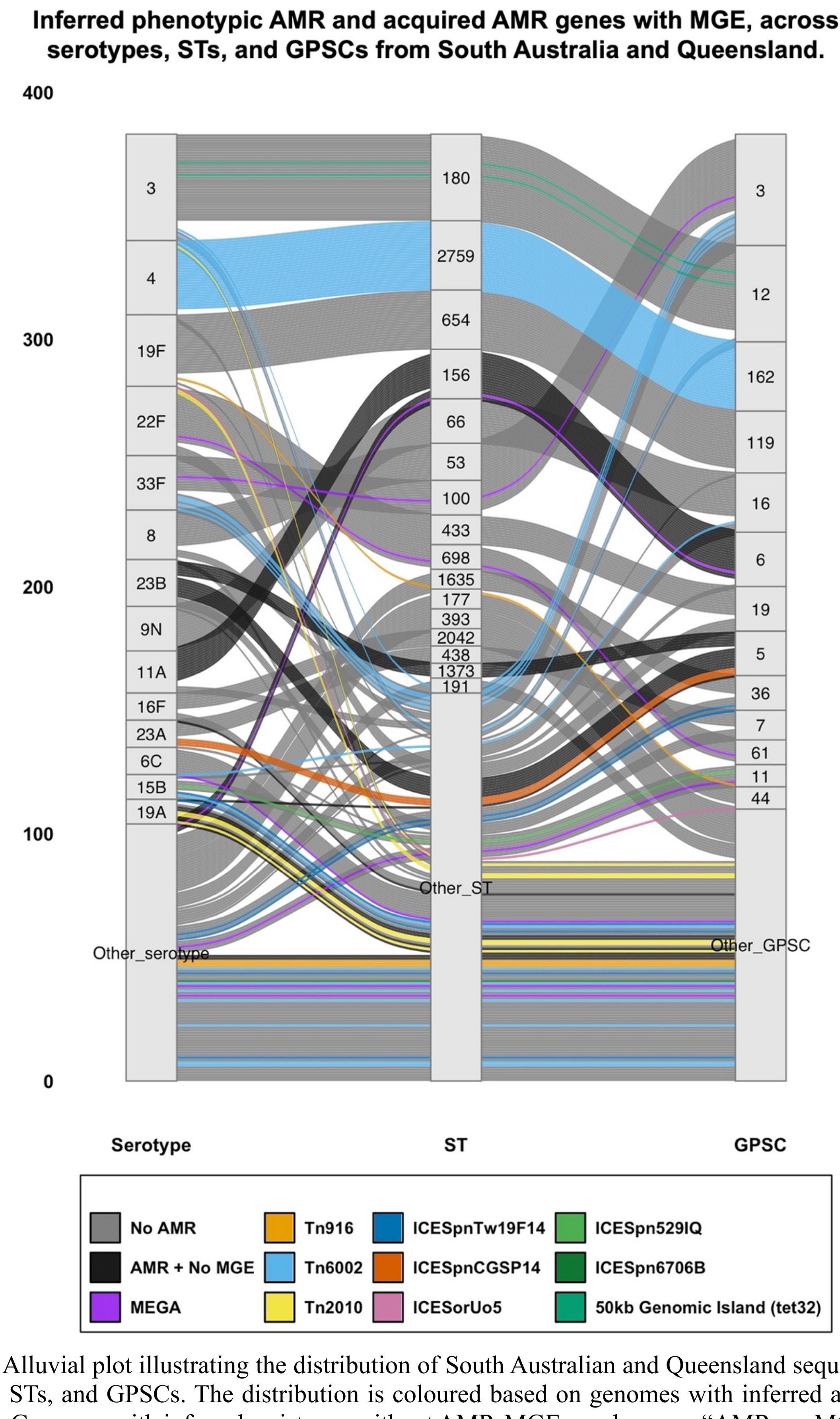

Fig. S1: Alluvial plot illustrating the distribution of South Australian and Queensland sequences across serotypes, STs, and GPSCs. The distribution is coloured based on genomes with inferred antimicrobial resistance. Genomes with inferred resistance without AMR-MGE are shown as "AMR, no MGE" (black), while genomes with no inferred resistance are shown as "no AMR" (grey). AMR-MGEs are represented as ICE*Sor*Uo5 (pink), ICE*Spn*529IQ (green), ICE*Spn*6706B (dark green), ICE*Spn*CGSP14 (orange), ICE*Spn*Tw19F14 (blue), MEGA (purple), Tn*2010* (yellow), Tn*6002* (light blue), Tn*916* (orange-yellow), and 50kb genomic island (tet32) (teal). Serotypes, STs, and GPSCs represented by fewer than 10, 6, and 9 sequences, respectively, are grouped as 'Other'.
